# Underwater Target Detection and 3D Reconstruction System Based on Binocular Vision

**DOI:** 10.3390/s18103570

**Published:** 2018-10-21

**Authors:** Guanying Huo, Ziyin Wu, Jiabiao Li, Shoujun Li

**Affiliations:** 1 Key Laboratory of Submarine Geosciences, Second Institute of Oceanography, State Oceanic Administration, Hangzhou 310012, China; jbli@sio.org.cn (J.L.); 0911guang@163.com (S.L.); 2 College of Internet of Things, Hohai University, Changzhou 213022, China

**Keywords:** underwater target detection, binocular vision, semi-global stereo matching, disparity map optimization, 3D reconstruction

## Abstract

To better solve the problem of target detection in marine environment and to deal with the difficulty of 3D reconstruction of underwater target, a binocular vision-based underwater target detection and 3D reconstruction system is proposed in this paper. Two optical sensors are used as the vision of the system. Firstly, denoising and color restoration are performed on the image sequence acquired by the vision of the system and the underwater target is segmented and extracted according to the image saliency using the super-pixel segmentation method. Secondly, aiming to reduce mismatch, we improve the semi-global stereo matching method by strictly constraining the matching in the valid target area and then optimizing the basic disparity map within each super-pixel area using the least squares fitting interpolation method. Finally, based on the optimized disparity map, triangulation principle is used to calculate the three-dimensional data of the target and the 3D structure and color information of the target can be given by MeshLab. The experimental results show that for a specific size underwater target, the system can achieve higher measurement accuracy and better 3D reconstruction effect within a suitable distance.

## 1. Introduction

With the rapid development of computer vision and robotics, now an enormous number of underwater operations can be conducted by underwater robots [1,2]. To succeed in autonomous underwater intervention, the integration process of the required robotic system is critical, which includes the mechatronics integration and the software integration. The mechatronics integration usually composes of three parts: underwater vehicle, robotic manipulator and a stereo vision system, while the software integration may include vehicle navigation, target identification, target tracking, arm control and visual control of the manipulator [3]. Underwater target detection and 3D reconstruction offered by the vision system, is a key issue for intervention missions carried out by underwater robots [4,5]. For intervention missions requiring grasping and manipulation of objects, the vision system must provide accurate target detection and distance estimation [6]. However, water not only influences the mechanical and electrical design of the robot sub-systems but also causes difficulties to the underwater vision. Water turbidity, color distortion, light absorption and scattering phenomena represent the major problems with underwater vision applications, which may affect the perceived features of the object and accordingly brings difficulties to underwater target detection and 3D reconstruction of the vision system [6]. Moreover, due to the flat-panel glass windows that are usually adopted for underwater vision housings, significant distortions due to refraction in air-glass-water transitions may happen and therefore an axial camera model may be more accurate than a pinhole model with the parameters obtained by an in-water calibration using a checkerboard [6,7]. 

In recent years, binocular stereo vision, which uses two cameras to get disparity map that can then be used to calculate the depth information and to achieve 3D reconstruction, has become popular in the field of computer vision [8]. Stereo matching, used for obtaining the disparity map, is a key issue and one of the most extensively studied problems in computer vision applications [9,10]. Stereo matching algorithms always have two major concerns: matching quality and computational efficiency [11,12,13]. Depending on whether the global search and refinement are performed or not, stereo matching algorithms can be divided into three categories: global, local and semi global [14]. The core of global matching algorithms is to define an energy function, which includes both data and smoothness energy terms [15,16,17]. Stereo matching can be regarded as an energy minimization problem and global disparity allocation can be obtained via optimization methods such as dynamic programming (DP) [18,19], graph cuts (GC) [20,21] and belief propagation (BP) [22,23], which are usually time-consuming and need substantial computational resources to achieve the optimal solution. The global methods could significantly improve the matching accuracy by a considerable computation cost [14,24]. The computation cost can be efficiently optimized by GPU-based methods, which could achieve higher processing speed. But porting existing techniques directly to GPU is also a cumbersome procedure because there are complex data structures and sequential processing [25,26,27]. Stereo algorithms based on local matching could overcome the afore-mentioned drawbacks. They can work much faster because they estimate pixel correspondence only within a small window [8,28]. However, the matching costs of local matching are more susceptible to noise. In a texture-less region, which contains minimal information, the costs of neighboring support regions are aggregated together, which may cause a less accurate result. In addition, the window size selection is a challenge as well [29,30]. Semi global matching (SGM) algorithm is known as a trade-off between accuracy and efficiency [31,32]. SGM methods adopt multiple paths optimization of disparity and achieve a minimum matching cost by the means of a winner-takes-all strategy based on hierarchical mutual information [33,34], which not only improves the calculation speed but also effectively solves the mismatch problem caused by the uneven illumination in images [35]. Therefore, it is a compromise strategy which is suitable for a real-time dense disparity map acquisition system based on binocular vision.

In this paper, aiming to reduce mismatch faced by underwater stereo matching, we mainly focus on further improving the SGM method by adopting two strategies: the first one is extracting the target area from the background with super-pixel segmentation and then constraining the matching within the valid target area and the second is optimizing the basic disparity map within every super-pixel area by the least squares fitting interpolation method. Based the improved SGM method, we also give a complete binocular stereo vision system that can be used by the underwater vehicles, which includes stereo calibration, image rectification, image denoising and color correction, image segmentation, stereo matching, depth calculation and 3D reconstruction. The proposed system can work well within a distance of 2 m. Firstly, the proposed system obtained the image sequence by high definition CMOS sensors on an underwater robot. Then, the image sequence is transmitted to the server through the Ethernet transmission module. And the data transmission between the server side and the PC end is realized by the wireless local area network (WLAN). After image rectification, denoising and color correction, the images are well restored and then the target areas in the left and right view images are detected by human visual attention mechanism and are segmented by the method of super-pixel clustering which is based on image saliency. After the segmentation, the stereo matching between the segmented areas are conducted by an improved SGM method. Moreover, an optimized disparity map was obtained by the least square plane fitting method. Finally, the depth information of the underwater targets was obtained by the principle of triangulation and a 3D model was reconstructed based on the three-dimensional coordinates. The experimental results show that the proposed method can achieve higher measurement accuracy and better 3D reconstruction effect.

## 2. The Underwater Target Detection and 3D Reconstruction System

### 2.1. Description of the System

**Figure 1:**
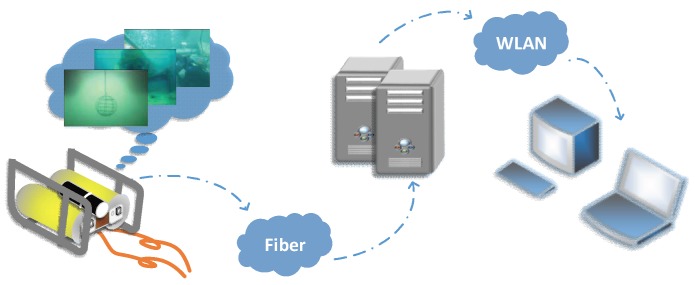
The Underwater Target Detection and 3D Reconstruction System.

The underwater target detection and 3D reconstruction system based on binocular vision proposed in this paper is shown in Figure 1. The whole system can be divided into two parts: perception module in underwater environment and data processing module on land surface. Under the normal operation of the system, the binocular vision sensing module, which is composed of two optical cameras, is used as the system input. The client of the data processing module on the land surface is set as the output of the system. The whole process mainly includes three steps: (1) Underwater target image sequence is collected by binocular vision system through video input interface module. (2) The collected image sequence is transmitted by the embedded subsystem through the data sending module, received by the data receiving module of the server and stored in the server’s memory. (3) The clients access the server’s memory through the Wireless Local Area Network (WLAN) to acquire the binocular image sequence and accomplish the image processing, which includes image pre-processing, stereo matching and 3D reconstruction. The disparity map and 3D coordinate information of targets are finally output by the system software interface.

### 2.2. Hardware of the System

**Figure 2:**
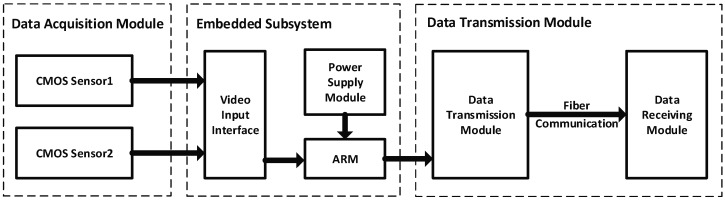
System hardware diagram.

As shown in Figure 2, the system hardware consists of three parts: data acquisition module, embedded subsystem and data transmission module. The CMOS Sensors (model FMVU-03MTC supplied by Lingliang Photoelectric Technology company in Shanghai, China) are adopted in this paper. Their resolution and frame are 640/480 and 60 fps, respectively. They are connected to the embedded subsystem through LVDS/HiSPi interface. The subsystem works based on HUAWEI Hass Hi3519V101 scheme, which uses ARM Cortex-A17 as the control core and supports video image acquisition of 1920 × 1080 30 fps and Gigabit Ethernet connections. The data transmission module uses optical fiber communication technology to transmit the collected image sequence data to the data receiving module of the server through the TCP/IP protocol.

### 2.3. Processing of The Binocular Vision System

**Figure 3:**
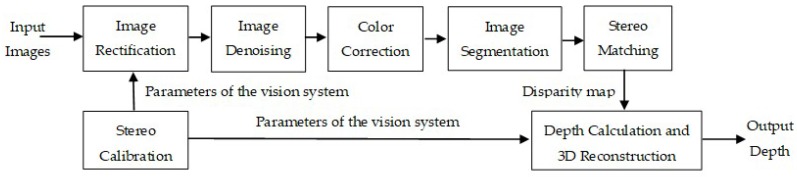
The binocular vision system processing flow chart.

As shown in Figure 3, the binocular vision system processing consists of stereo calibration, image rectification, image denoising, color correction, image segmentation, stereo matching, depth calculation and 3D reconstruction. Stereo calibration is done off-line by an in-water calibration using a checkerboard in our current work, which will be described in Section 3.1.1. Image rectification projects the left and right images onto a common plane in such a way that the corresponding points have the same row coordinates and will be given in Section 3.1.2. Image denoising and color correction, offered in Section 3.2.1 and Section 3.2.2 respectively, together improve the quality of underwater images for better image segmentation and stereo matching. Image segmentation in Section 3.2.3 is used for target extraction and matching area restriction. Stereo matching, which is described in Section 3.2.4, can produce the disparity map, which will then be used for depth calculation and 3D reconstruction. Based on the above processing, we have developed a software of the binocular vision system using the Microsoft Foundation Class (MFC) library and C++ language. The user can view both left and right images in the current frame, obtain the disparity map and depth information of the target and view the 3D scene of the target if necessary.

## 3. Methods and Implementation

### 3.1. Off-Line Calibration of the Binocular Vision System

#### 3.1.1. Stereo Calibration

Stereo measurement based on binocular vision has the similar principles as triangulation technology. The target images are obtained by two cameras from different angles. Then the 3D geometric information is acquired from the 2D image coordinates of the feature points in the left and right views. The calibration accuracy of binocular stereo vision system is an important factor affecting the accuracy of 3D reconstruction. The fundamental task of calibration of stereo vision system is to determine the mapping relation between the 2D image coordinates and the 3D world coordinates, including the optical geometry parameters and distortion parameters (intrinsic parameters) that describe the internal structure of each camera and the structural parameters (external parameters) that describe the spatial relationship between the two cameras. Due to light refraction in air-glass-water transitions, an axial camera is more suitable for modeling an underwater camera [6,7]. However, an axial camera model is still difficult to use with stereo processing and therefore it is still common in underwater vision to adopt a pinhole model with the parameters obtained by an in-water calibration using a checkerboard [6]. The calibration method based on the chessboard template proposed by Zhang [36] has a higher calibration precision and is widely used. According to the convenience and maneuverability, the proposed system currently uses Zhang’s chessboard calibration method to restrict the intrinsic parameters of cameras through the corner feature and the homographic matrix. The mapping relation between the 3D world coordinate system and the 2D pixel coordinate system is defined as follows:

*s∙p* = *M* [*R T*]∙*P* (1)

where *P* = [*X_w_ Y_w_ Z*_w_ 1] is the homogeneous coordinate of a given point in the world coordinate system; *p* = [*u v* 1] is the corresponding homogeneous coordinate of this point in the pixel coordinate system; *s* is a scaling factor; *R* is a rotation matrix and *T* is a translation vector; *M* is the camera intrinsic matrix and can be given by the following:

(2) $$M=\left[\begin{array}{ccc} f_{x} & 0 & u_{0}\\ 0 & f_{y} & v_{0}\\ 0 & 0 & 1 \end{array}\right]$$

where *f_x_* = *f*/*d_x_*, *f_y_* = *f*/*d_y_*; *d_x_* and *d_y_* are the sizes of a single pixel in the direction of the *X* axis and that of the *Y* axis of the image coordinate system respectively; (*u*_0_, *v*_0_) is the position of origin point of pixel coordinate system in the image coordinate system. If a point is at the position (*u*, *v*) in the pixel coordinate system and at (*x*_c_, *y*_c_) in the image coordinate system, the corresponding coordinate relations will be given by the following:

(3) $$\left\{ \begin{array}{l} u=\frac{x_{c}}{d_{x}}+u_{0}\\ v=\frac{y_{c}}{d_{y}}+v_{0} \end{array}\right.\;$$

The above solving process of camera intrinsic parameters assumes that the lens is an ideal model. The image is usually distorted by the influence of the lens manufacturing process. According to Brown’s theory for distortion parameter solution, the lens distortion can be described by radial distortion and tangential distortion and the expression is as follows:

(4) $$\left[\begin{array}{l} x_{rec}\\ y_{rec} \end{array}\right]=\left(1+k_{1}r^{2}+k_{2}r^{4}+k_{3}r^{6}\right)\left[\begin{array}{l} x\\ y \end{array}\right]+\left[\begin{array}{l} 2p_{1}xy+p_{2}(r^{2}+2x^{2})\\ p_{1}(r^{2}+2y^{2})+2p_{2}xy \end{array}\right]\;$$

where (*x*, *y*) is the original position of the distortion point and (*x_rec_*, *y_rec_*) is the new position after correction; *r* is the radius of the lens model; *k*_1_, *k*_2_ and *k*_3_ are radial distortion parameters; $p_{1},p_{2}$ are tangential distortion parameters; and *D* = [*k*_1_, *k*_2_, *k*_3_, *p*_1_, *p*_2_] is the distortion parameter vector of the non-ideal lens model.

The external parameters of the binocular camera include the rotation matrix *R*, the translation vector *T* and the re-projection matrix *Q*. The rotation matrix *R* and the translation vector *T* are used to describe the relative position of the binocular cameras and can be given by:

(5) $$\left\{ \begin{array}{l} R=R_{r}{\left(R_{l}\right)}^{T}\\ T=T_{r}-RT_{l} \end{array}\right.\;$$

where *R_r_* and *T_r_* are the rotation matrix and the translation vector for the right camera; while *R_l_* and *T_l_* are those for the left one. The re-projection matrix *Q* is used to convert the two-dimensional image point coordinates into three-dimensional coordinates, which can be calculated according to the following:

(6) $$Q=\left[\begin{array}{cccc} 1 & 0 & 0 & -c_{x}\\ 0 & 1 & 0 & -c_{y}\\ 0 & 0 & 0 & f\\ 0 & 0 & -1/T_{x} & \left(c_{x}-c_{x}^{\prime }\right)/T_{x} \end{array}\right]\;$$

where (*c_x_*, *c_y_*) is the origin position of the left view image and $c_{x}{}^{\prime }$ is the horizontal ordinate of the right view image origin; *T_x_* is the component of the translation matrix in the direction of the *X* axis, which equals the baseline distance *B* between the binocular cameras in the ideal situation; and *f* is the focal length of the lenses. To measure the calibration accuracy, the error of the calibration result is characterized by calculating the re-projection error of the corner point of the checkerboard, which is defined as:

(7) $$err=\frac{1}{n}\sum_{i=1}^{n}\sqrt{{\left(u_{1i}-u_{2i}\right)}^{2}+{\left(v_{1i}-v_{2i}\right)}^{2}}\;$$

where $\left(u_{1i},v_{1i}\right)$ is the sub-pixel corner coordinates extracted from the feature points; $\left(u_{2i},v_{2i}\right)$ is the coordinates calculated according to the re-projection matrix; and *n* is the total number of calibration checkerboard corners.

After stereo calibration using a checkerboard with 1-m distance in water and in the air, intrinsic parameters of the binocular vision system in water are given in Table 1 and the corresponding intrinsic parameters in the air are given in Table 2. According to Table 1 and Table 2, the average focal length ratio in water and in the air can be calculated by

(8) $$\alpha_{avg}=\frac{1}{4}\left(\frac{f_{x\_water\_L}}{f_{x\_air\_L}}+\frac{f_{x\_water\_R}}{f_{x\_air\_R}}+\frac{f_{y\_water\_L}}{f_{y\_air\_L}}+\frac{f_{y\_water\_R}}{f_{y\_air\_R}}\right)\approx 1.357\;$$

which is a little bigger than the ideal focal length ratio of 1.333. External parameters that gives the relative spatial position between the two optical sensors are given in Table 3. Besides, the calibration errors of 0.173 (unit: pixel) in water and 0.155 (unit: pixel) in the air are also given in Table 3. From Table 3, it can be seen that in water, the external parameters are relatively small, that is, the binocular images in water can be corrected by the rotation of a smaller angle and the translation of a smaller distance than in the air.

#### 3.1.2. Image Rectification

**Figure 4:**
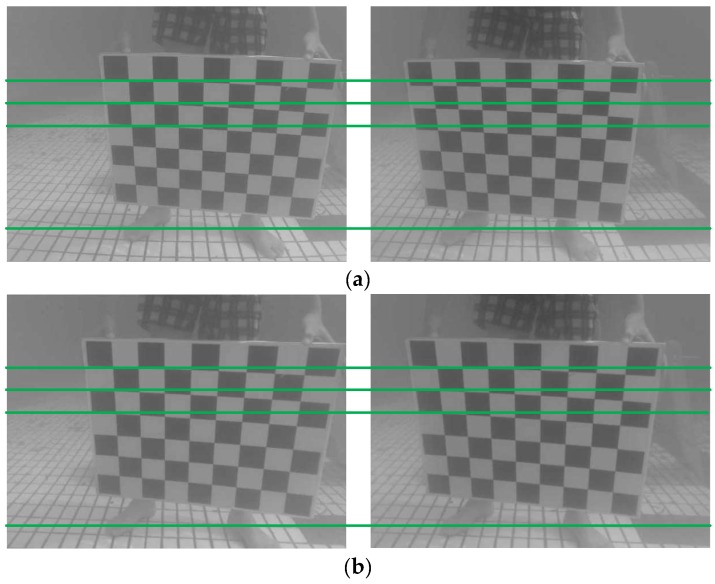
Comparison of the left and right images before and after correction. (**a**) The left and right images before correction; (**b**) The left and right images after correction.

The left and right images obtained by the binocular vision system usually have a certain image distortion due to the imaging principle of the camera and the structure of the device. Therefore, the same pixel may not be on the same pole line in the left and right images. This will bring difficulties to the subsequent stereo matching and cause the increase in time consumption and mismatch. To improve the accuracy of stereo matching, it is necessary to correct the image to achieve the strict coplane and line alignment of the left and right images in theory. The search range of image matching points is reduced from two-dimensional to one dimension, so the range of search is reduced and the speed of the operation can be improved. Image correction includes three steps: elimination of distortion, image correction and image cutting. The proposed system made use of OpenCV Library. Firstly, the ‘pillow’ distortion is eliminated based on the distortion parameters obtained from the calibration process in Section 3.1.1. Secondly, the left and right view images are horizontally aligned by parameters with the focal length and optical center of lens, rotation matrix and translation vector. Therefore, we can ensure the position consistence of optical centers, parallelism of light axis and alignment of polar lines. Finally, the image is cut and the irregular areas at the edges and corners of the image are deleted so that the overlapped area of the left and right images is maximized. The rectification results of the checkboard images are shown in Figure 4.

As shown in Figure 4a is the image pair before image correction and Figure 4b is the image pair after image correction. Obviously, the ‘pillow’ distortion is eliminated by stereoscopic correction and the corresponding points of the same targets in the left and right images are basically aligned.

### 3.2. Implementation of 3D Reconstruction System

#### 3.2.1. Image Denoising

Obtaining high-quality underwater images is important for accurate 3D reconstruction of underwater targets. To effectively remove noise in the images, the proposed system adopts the block matching and 3D filtering (BM3D) algorithm [37]. The BM3D algorithm can be divided into two steps: (1) Initial estimation and (2) Final estimation. In the first step, the similarity between search block and reference block is defined by a hard threshold. The similar blocks are stacked into three-dimensional arrays. After each three-dimensional array is filtered through cooperative filtering, that is, the spectrum transform is contracted, the initial estimate of the reference block is obtained by inverse transformation. Finally, the initial estimation is aggregated by a method of the non-local mean. In the second process, the original image and the initial estimation image are transformed by three-dimensional transform and Wiener filtering and the final output is obtained by inverse transform and aggregation.

#### 3.2.2. Color Correction

Due to the limitations of the digital camera’s photosensitive device, there is a difference between the recorded color and the real color. This difference can be expressed as a relation of mapping. Hirschmuller [33] estimated the downlink attenuation coefficients of different colors under the water based on the multispectral or hyperspectral data of underwater images of target at a specific location. By reversely creating an unenhanced image, the over-enhanced phenomenon caused by the camera built-in functions of white balance and color enhancement is eliminated. Therefore, the color information of the underwater image is effectively restored. The proposed system used three basic color (RGB) calibration method to calibrate and restore the color information of images. Through a variety of curve fitting tests, it is found that the three polynomial fitting results are the best and the fitting formula of the photosensitive curve is as follows:

(9) $$\left\{ \begin{array}{l} R=A_{r}r^{3}+B_{r}r^{2}+C_{r}r+D_{r}\\ G=A_{g}g^{3}+B_{g}g^{2}+C_{g}g+D_{g}\\ B=A_{b}b^{3}+B_{b}b^{2}+C_{b}b+D_{b} \end{array}\right.\;$$

where *r*, *g* and *b* are recorded values of digital cameras for red, green and blue, respectively; *R*, *G* and *B* are standard values for red, green and blue. We carried out the color correction experiment with ‘ColorChecker 24’ using the method of three basic color calibration and achieved the fitting coefficient of the photoreceptor curve. The photoreceptor curve fitting parameters of cameras are given in Table 4.

**Figure 5:**
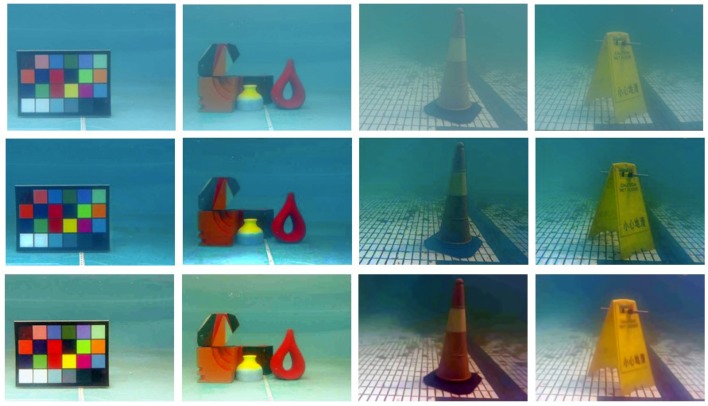
Comparison of the original underwater images, the results using the method in Reference [38] and the results using BM3D filtering and color correction, where the first row shows the original underwater images, the second row shows the results using the method in Reference [38] and the third row shows the results using BM3D filtering and color correction.

**Figure 6:**
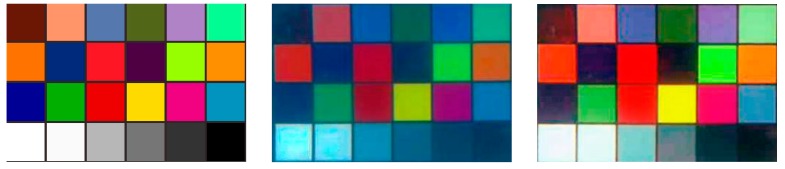
Comparison of the standard color checkboard, the color checkboard restored by the method in Reference [38] and that restored by BM3D filtering and color correction, which are shown from left to right.

To demonstrate the effectiveness of our proposal, Figure 5 gives a comparison of the original underwater images, the results using the dark channel prior method in Reference [38] and the results using BM3D filtering and color correction of our research. It can be seen from Figure 5 that, the ‘atomization’ phenomenon is effectively eliminated and the true color information of the image is well restored after BM3D filtering and color correction. By using BM3D filtering and color correction, the targets in the underwater images become much clearer, which will contribute to the following accurate stereo matching of the image pairs. Compared with our method, although the method in Reference [38] can also well remove the ‘atomization’ phenomenon, it may fail to recover the true color, which can be seen from the first three images in the second row. In the first image of the second row of Figure 5, the third and fifth blocks, which should have different colors, are made the same color; and that is the same case for the square target and vase in the second image, which should have different red colors; while in the third image, the red color is not well recovered. To clearly demonstrate this, the standard color checkboard, the color checkboard restored by the method in Reference [38] and that restored by BM3D filtering and color correction are further shown in detail in Figure 6, from which it can be seen that the color checkboard restored by BM3D filtering and color correction is much closer to the real one.

#### 3.2.3. Image Segmentation

After image denoising and color restoration, the true colors of images are obtained. However, due to the particularity of marine environment, the background of underwater images usually contains less texture information, which may cause many mismatched regions. Therefore, the proposed system implemented the segmentation and extraction of the target from the background before the stereo image matching. Taking the advantages of the super-pixel segmentation algorithm that can reduce the complexity of the subsequent image processing, a segmentation algorithm based on super-pixel clustering is adopted in this paper. First, the brightness and texture features are extracted from the underwater image after the noise reduction. Next, the similarity of the two features is calculated to make a weighted fusion. Then, the pixels are clustered to generate the super-pixels by using the fusion similarity as the distance measurement. The calculation formula of the distance metric *D* is as follows:

(10) $$D=\lambda \cdot d_{t}+(1-\lambda )\sqrt{d_{c}^{2}+{\left(\frac{N_{c}}{N_{s}}\cdot d_{s}\right)}^{2}}\;$$

where *d_t_*, *d*_c_ and *d*_s_ are similarity distances for texture, color and spatial features, respectively; *N*_s_ is the maximum space distance within the cluster; *N*_c_ is the maximum color similarity; and λ is a weight parameter. Obviously, the smaller the distance metric *D* is, the greater the similarity between the pixels.

**Figure 7:**
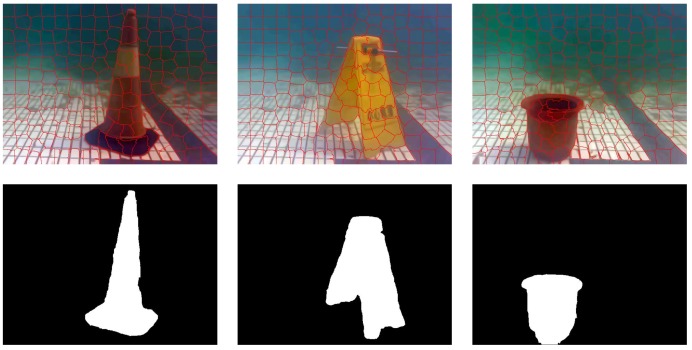
Segmentation results of targets (super-pixels number: 200).

Figure 7 shows the results of the target segmentation. Based on the feature, the generated super-pixels are detected and the super-pixels with saliency are marked by red line. All the super-pixels are clustered by the method of the Max-Flow/Min-Cut algorithm. After that, the proportion of the significant super-pixels in clustering is calculated and compared with the preset threshold. Thus, the segmentation result for the foreground object is obtained.

#### 3.2.4. Stereo Matching

**Figure 8:**
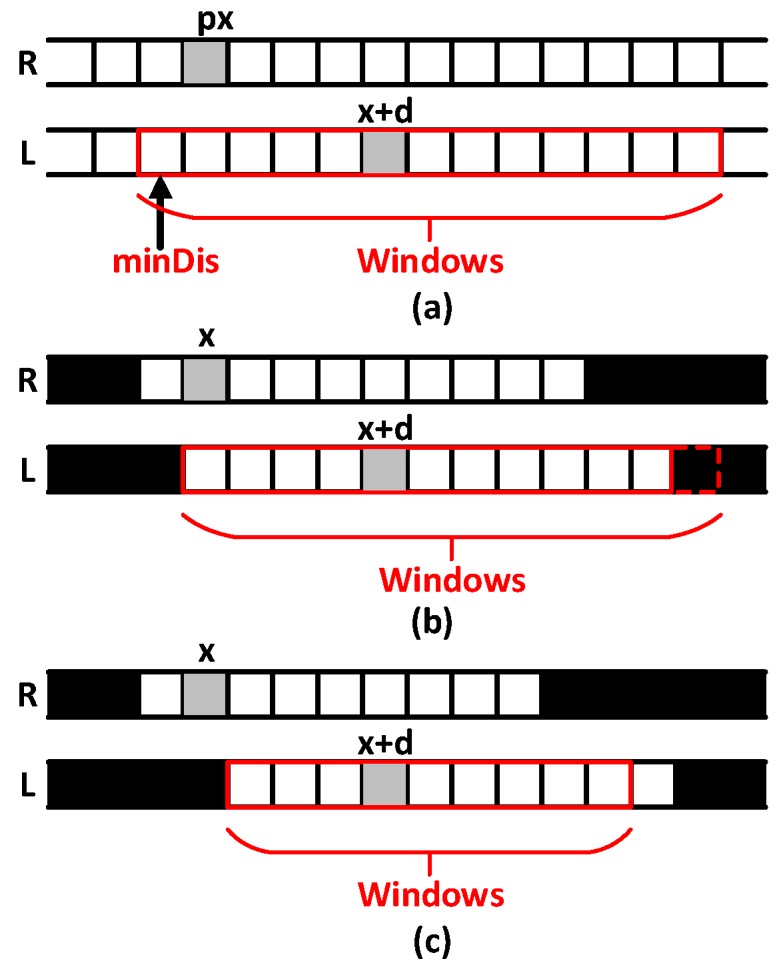
Comparison of disparity calculation. (**a**) The stereo matching principle of SGM algorithm; (**b**) Stereo matching without constraint of the valid target area; (**c**) Stereo matching strictly constrained within the valid target area.

Stereo matching is an important part of the system implementation. The system uses binocular cameras to get different views of the left and right images, to calculate the cost of stereo matching and get the matching disparity map. Considering both matching accuracy and time efficiency, the Semi-Global Matching (SGM) algorithm that has the advantages of fast matching speed and high matching accuracy is preferred. According to this, we proposed an improved algorithm based on the semi-global matching algorithm in our system. Taking the right view as the reference, Figure 8a shows the stereo matching principle of SGM algorithm. If there is a point to be matched in the right view image and its horizontal ordinate is *x*, we could search for the best match point starting from the position *minDis* within the range of *Windows* in the left view image. However, the disadvantage of this algorithm is that the color characteristics of underwater images are seriously disturbed and degraded by the influence of light and water scattering. Therefore, there are many mismatches in the background areas.

The improved stereo matching algorithm based on SGM algorithm proposed in this paper can accurately extract the target area from the background in underwater images. The stereo matching process is strictly constrained within the valid target area. As shown in Figure 8b,c, the black pixels belong to background that are invalid for stereo matching, while the white ones belong to target areas that are valid for stereo matching; and the gray one is the current pixel to be matched. If the matching pixel in the left image of the current pixel to be matched in the right one is in the valid target area, the matching search process starts directly from the position *x* in the left image until the matching pixel reaches the boundary of search window. If the boundary of search window is in the invalid background area, the search process ends in advance. If the matching pixel in the left image of the current pixel to be matched in the right one is in the invalid background area, the matching search process starts from the first valid pixel in the search window until the matching pixel reaches the window boundary or the invalid background. The implementation of the improved algorithm can be divided into the following four steps:

(1) Gradient information extraction. To further eliminate the effect of image noise on calculation of disparity map, the horizontal Sobel operator is used to extract the gradient information of the image. The Sobel operator is given as follows:

(11) Sobel(x,y)=2[I(x+1,y)−I(x−1,y)]+[I(x+1,y−1)−I(x−1,y−1)]+[I(x+1,y+1)−I(x−1,y+1)] 

where *I* represents the pixel value of the image. After processed by the Sobel operator and smoothed by the Gauss filter, the original image is mapped to generate a new image. The mapping function is given by:

(12) $$I_{new}=\left\{ \begin{array}{cc} 0\;, & I<-T_{h}\\ I+T_{h}, & -T_{h}\leq I\leq T_{h}\\ 2*T_{h}\;, & I>T_{h} \end{array}\right.\;$$

where *I* represents the pixel value of the original image; *I_new_* indicates the pixel value of the remapping image; and *T_h_* is the threshold of the filter.

(2) Matching cost calculation. In practical applications, the different visual angle of the binocular vision system always leads to an inhomogeneous phenomenon between the left and right view images, which causes an increase in the mismatch rate. Mutual information has the advantage of insensitivity to light, so the semi-global matching algorithm is based on such information. The computational efficiency and accuracy of the stereo matching are improved by the cost calculation of the hierarchical mutual information instead of the traditional gray value calculation. The definition of mutual information is as follows:

(13) $$M_{I_{1},I_{2}}=H_{I_{1}}+H_{I_{2}}-H_{I_{1},I_{2}}\;$$

where $H_{I_{1}}$ and $H_{I_{2}}$ are the entropies of the left and right images, respectively; $H_{I_{1},I_{2}}$ is the combined entropy for the two images. According to the Taylor expansion formula, entropy $H_{I}$ and combined entropy $H_{I_{1},I_{2}}$ can be respectively expressed as:

(14) $$\left\{ \begin{array}{l} H_{I}=\sum_{p}h_{I}(I_{p})\;,\;h_{I}(i)=-\frac{1}{n}\log\left(P_{I}(i)⊗g(i)\right)⊗g(i)\\ H_{I_{1},I_{2}}=\sum_{p}h_{I_{1},I_{2}}(I_{1p},I_{2p})\;,\;h_{I_{1},I_{2}}(i,k)=-\frac{1}{n}\log\left(P_{I_{1},I_{2}}(i,k)⊗g(i,k)\right)⊗g(i,k)\; \end{array}\right.\;$$

where $P_{I_{1},I_{2}}(i,k)$ represents the joint probability distribution of the images; *g*(*i*, *k*)is the Gauss kernel function. Therefore, the mutual information $M_{I_{1},I_{2}}$ can be finally given by:

(15) $$M_{I_{1},I_{2}}=\sum_{p}m_{I_{1},I_{2}}(I_{1p},I_{2p}){\;,\;m}_{I_{1},I_{2}}(i,k)=h_{I_{1}}(i)+h_{I_{2}}(k)-h_{I_{1},I_{2}}(i,k)\;$$

Then, the corresponding matching cost is defined as:

(16) $$C\left(p,d\right)=-m_{I_{p},I_{q}}\left(I_{p},I_{q}\right)\;$$

where *I*_p_ is the value of point p and q is its corresponding point on the polar line in the left view image. If the horizontal ordinate of p is *x*, then the horizontal ordinate of q is *x* + *d*, where *d* is the disparity value.

(3) Cost aggregation. The matching cost based on the mutual information has been obtained after the above calculation process for matching cost. But such matching cost with the form of pixel by pixel can be easily affected by mismatch points or noise and other factors. Therefore, the penalty function based on the neighborhood disparity data is introduced to increase the smoothness constraint. Accordingly, the energy function can be defined as:

(17) $$E\left(d\right)=\sum_{p}C\left(p,d_{p}\right)+\sum_{q\in {Ν}_{p}}P_{1}T\left[\left|d_{p}-d_{q}\right|=1\right]+\sum_{q\in {Ν}_{p}}P_{2}T\left[\left|d_{p}-d_{q}\right|>1\right]\;$$

where $\sum_{p}C\left(p,d_{p}\right)$ is the data item representing the matching costs of all pixels in the image and the next two items are used for punishment. If the disparity value between the point p and the point q equals 1, the punishment item *P*_1_ works; otherwise if the disparity value is greater than 1 and *P*_2_ is larger than *P*_1_ at the same time, the punishment item *P*_2_ works. Besides, q is a point within the neighborhood (${Ν}_{p}$) of point p. To minimize the energy, the dynamic programming method is adopted and the idea of scanning line optimization is introduced. The matching cost on the direction *r* could be defined as:

(18) $$L_{r}\left(p,d\right)=C\left(p,d\right)+\min\left(\begin{array}{l} L_{r}\left(p-r,d\right),\\ L_{r}\left(p-r,d-1\right)+P_{1},L_{r}\left(p-r,d+1\right)+P_{1},\underset{i}{\min}L_{r}\left(p-r,i\right)+P_{2},\\ \underset{k}{\min}L_{r}\left(p-r,k\right) \end{array}\right)\;$$

where C(p,d) indicates the matching cost of point p on disparity value *d*; the second term represents the minimum matching cost of the path adjacent point p−r based on the disparity smoothing constraint; and the third term represents the minimum matching cost of the path adjacent point p−r along the direction *r*. Therefore, the sum of the matching costs of point p can be obtained by aggregation of the path costs in the direction of each scan line, which is given by Equation (18):

(19) $$S\left(p,d\right)=\sum_{r}L_{r}\left(p,d\right)\;$$

(4) Disparity map optimization. According to the above matching cost calculation method, the right view image was set as the reference and the left view image was the one to be matched. The effective area of the whole image is traversed by progressive scanning. Upon every valid pixel in the right view getting the best matching point with the lowest matching cost in the left view, the basic disparity map was consequently formed. Aiming at the problem of mismatching or invalid disparity in the weak texture area, the proposed method made use of the super-pixel segmentation data to optimize the basic disparity map within every super-pixel area by the least squares fitting interpolation method. The plane template used in this paper is given by Equation (20):

(20) d(x,y)=ax+by+c 

The weighted least square method was used to calculate *a*, *b* and *c*, which formed the parameters set of the disparity plane template. The calculation formula of the weighted least square method is as follows:

(21) $$\left[\begin{array}{ccc} \sum x_{i}^{2} & \sum x_{i}y_{i} & \sum x_{i}\\ \sum x_{i}y_{i} & \sum y_{i}^{2} & \sum y_{i}\\ \sum x_{i} & \sum y_{i} & N \end{array}\right]{\left[\begin{array}{ccc} a & b & c \end{array}\right]}^{T}=\left[\begin{array}{c} \sum x_{i}d_{i}\\ \sum y_{i}d_{i}\\ \sum d_{i} \end{array}\right]\;$$

where *N* is the total number of pixels in the plane area; (*x_i_*, *y_i_*) and *d_i_* are the coordinates and disparity values of the pixel indexed by *i*, respectively.

**Figure 9:**
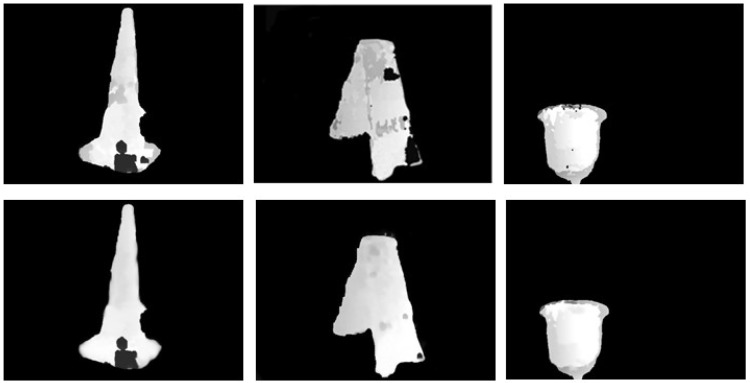
Optimization results of the disparity maps, where the basic disparity maps and the optimization results of the least squares plane fitting interpolation method are given in the first row and second row, respectively.

As shown in Figure 9, compared with the basic disparity maps obtained by stereo matching, the optimization results of the least squares plane fitting interpolation method are smoother and more complete, with fewer holes. The invalid matching areas have been basically eliminated. In addition, the disparity plane in the same area is effectively smoothed and the transition of disparity values is more placid. The fitting parameters of the disparity plane templates obtained for optimizing the three basic disparity maps are given in Table 5.

**Figure 10:**
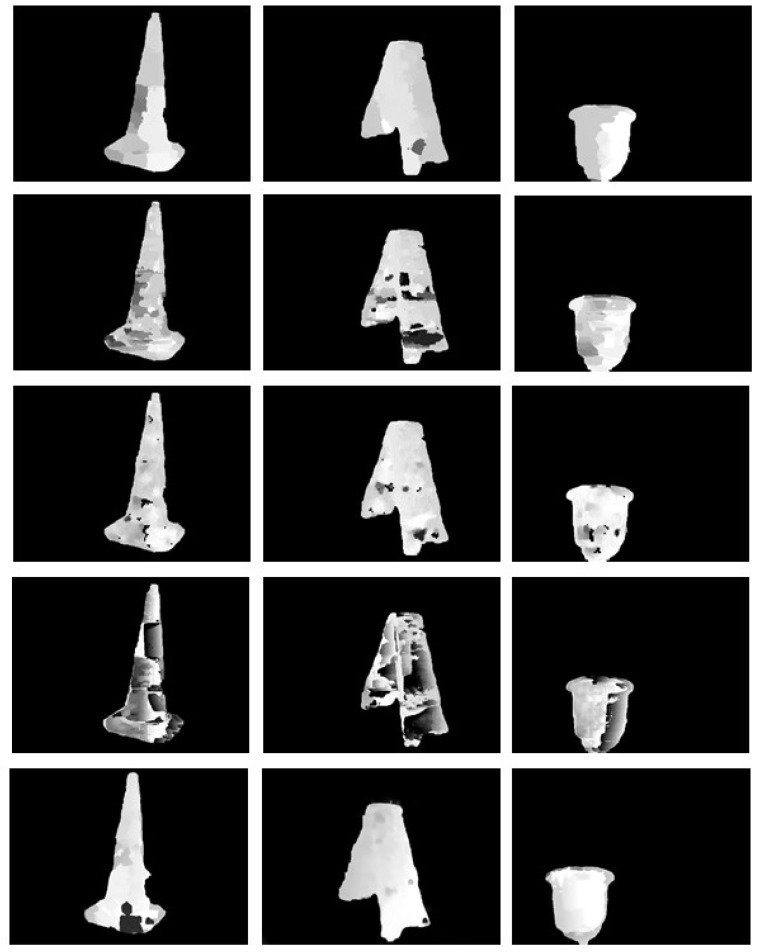
Comparison of the disparity maps produced by the AD-Census method, the FCVF method, the ARWR method, the SGM method and the proposed method, from top to bottom.

In Figure 10, we provide a comparison of the disparity maps produced by our method and four state of the art stereo matching methods, which are AD-Census method by Mei et al., Fast Cost-Volume Filtering (FCVF) method by Hosni et al., Adaptive Random Walk with Restart (ARWR) method by Lee et al. and Semi Global Matching (SGM) method by Hirschmuller et al. Among the five methods, the proposed method usually can provide best disparity maps, which are smooth and continuous, with fewer black holes. The two global matching methods, that is, the AD-Census method and the ARWR method, have better performance than the FCVF method and the SGM method. By constraining the matching within the valid target area and further optimizing the basic disparity map using the least squares plane fitting interpolation, the proposed method has made a remarkable improvement of the SGM method. 

## 4. Results and Discussion

Based on the disparity map, the depth information that gives the distance from the target to the camera can finally be calculated. In this section, we first give the measurement principle of binocular vision and then provide a comparison of the distance measurement accuracy using different disparity maps produced by the five stereo matching methods mentioned above. The 3D reconstruction results using different disparity maps are also shown here.

**Figure 11:**
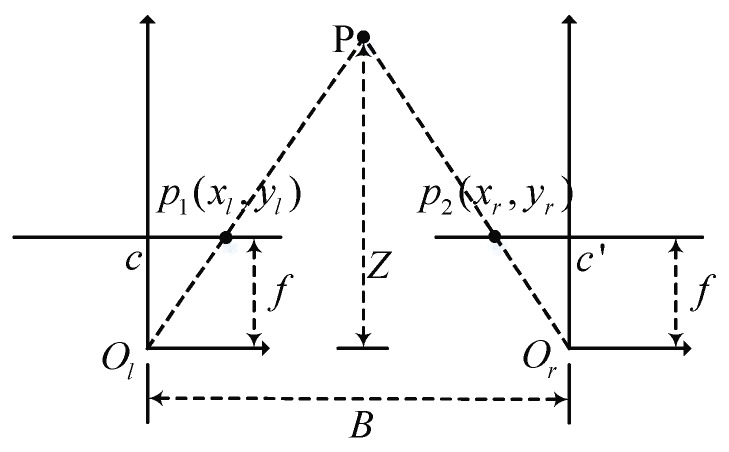
Diagram of binocular vision measurement principle.

Figure 11 shows the measurement principle of binocular vision. Assuming the imaging planes of binocular cameras are coplanar and the distances to the camera’s optical centers ($O_{l}$ and $O_{r}$) are *f*. The baseline distance between two camera optical centers is *B*. If the objective point P is located at *p*_1_(*x_l_*, *y_l_*) in the left view image and at *p*_2_(*x_r_*, *y_r_*) in the right view image, then the distance *Z* from the objective point P to the imaging plane, also known as the depth value, can be calculated according to the following:

(22) $$\begin{array}{c} \frac{B}{Z}=\frac{B-\left[\left(x_{l}-c_{x}\right)+\left(c_{x}^{\prime }-x_{r}\right)\right]}{Z-f}\\ ⇓\\ Z=\frac{f\cdot B}{d-\left(c_{x}-c_{x}^{\prime }\right)} \end{array}$$

where *d* represents the disparity value; $c_{x}$ and $c_{x}^{\prime }$ are the horizontal ordinates of the center points of the two imaging planes. In addition to the depth value, we can also use the reprojection matrix computed in the previous stereoscopic calibration process to get the horizontal and vertical coordinates of the objective point P in *X*-axis and *Y*-axis directions. Based on the dense disparity maps, we can transform 2D coordinate into 3D coordinate information according to the following:

(23) $${\left[\begin{array}{cccc} X & Y & Z & W \end{array}\right]}^{T}=Q{\left[\begin{array}{cccc} x & y & d & 1 \end{array}\right]}^{T}\;$$

where (*x*, *y*) is the pixel coordinate of a point in the disparity map and *d* is the disparity value of the point. Then, the real-world coordinate of the point can be expressed as $\left(\frac{X}{W},\frac{Y}{W},\frac{Z}{W}\right)$.

**Figure 12:**
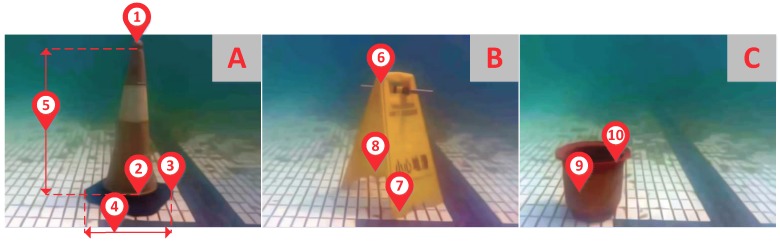
Sampling points of our measurement experiments. (**A**) The underwater image of a barrier; (**B**) The underwater image of a warning notice; (**C**) The underwater image of a flowerpot.

To evaluate the accuracy and precision of the proposed method, we carried out a series of distance measurement experiments. We sampled several points from different underwater targets and obtained 10 distance values measured by our method and the compared methods, separately. According to the implementation, we set a valid distance range of measurement within [0, 200]. And the distance unit is centimeter. The sampling points positions are shown in Figure 12. Sampling points’ pixel coordinates are (380, 40), (380, 385), (460, 400) and (250, 400) in image A; (350, 120), (390, 450) and (340, 335) in image B; (200, 380) and (300, 310) in image C.

In our implementation, all experiment data are obtained in Intel (R) Core (TM) i5-2467M processor, 4 GB memory, CPU frequency 1.6 GHz, Windows 7 system PC machine, Visual Studio 2015. Table 6 shows the data of measurement experiments for underwater targets. As known from Table 6, all the values of sampling points are valid. The proposed method has the lowest error rate (mean error of 2.320%) compared with the other four methods and has made a great progress of SGM algorithm (mean error of 10.114%).

**Figure 13:**
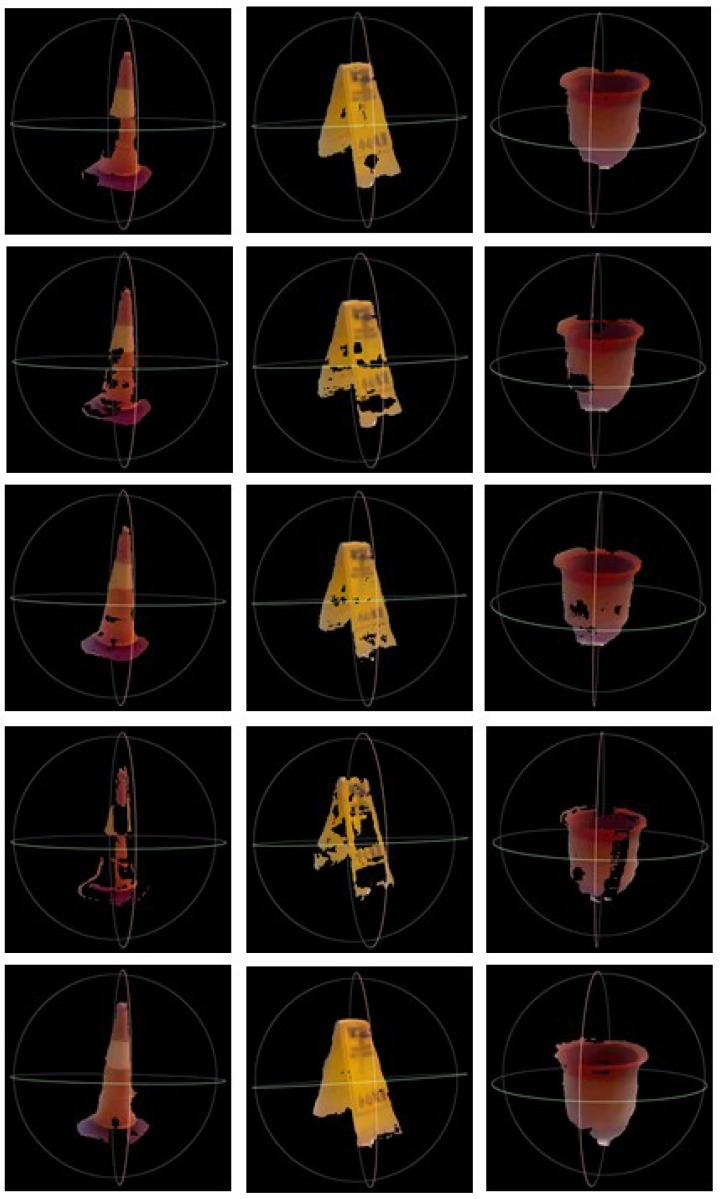
Comparison of 3D reconstruction of underwater targets using disparity maps provided by methods of AD-Census method, the FCVF method, the ARWR method, the SGM method and the proposed method, which are demonstrated from the first row to the last row, respectively.

To display the structure information of the underwater targets more intuitively and effectively, the 3D scene can be reconstructed. Figure 13 gives the results of AD-Census method, the FCVF method, the ARWR method, the SGM method and the proposed method, respectively. The proposed system adopts the open source and extensible 3D geometric processing software MeshLab to reconstruct the color information and 3D spatial data of the targets. Figure 13 effectively demonstrates the accuracy of the system for underwater target detection and 3D reconstruction.

From Table 6 and Figure 13, it can be seen that:

(1) Among the four methods compared with our method, the ARWR and AD-Census methods have better performance than the other two because their matching cost computations are both global and have taken account of the overall scene structure. As shown in Table 6, they can produce more accurate distance measurement results than the other two, with mean error rates at 3.553% and 6.752%, respectively. But meanwhile, they reduce the computational efficiency of the system.
(2) The SGM method has a trade-off between accuracy and computation requirements, which is suitable for practical applications. However, many invalid matches are made on the surface where there is a large area or a smooth changing, which can be clearly seen in Figure 13e. This is because that considering pixel correspondence only within a small window are more susceptible to noise and that the costs of neighboring support regions in the texture-less area are aggregated together to give a less accurate result.
(3) The proposed method is based on the SGM method and therefore it inherits the advantage of high efficiency. For the above testing images, the average execution time of the proposed method is about 90 milliseconds, which is close to the average execution time of the SGM method at 70 milliseconds. The FCVF method is the fastest one with an average 40 milliseconds. While the AD-Census method and the ARWR method cost about 600 milliseconds and 5 s, respectively. Moreover, by strictly constraining the stereo matching in the valid target area and optimizing the basic disparity map within each super-pixel area using the least squares fitting interpolation method, the accuracy of measurement can be greatly improved. Therefore, the proposed method has the lowest error rate (mean error of 2.320%) among all the five methods.

## 5. Limitations of the Proposed Method

**Figure 14:**
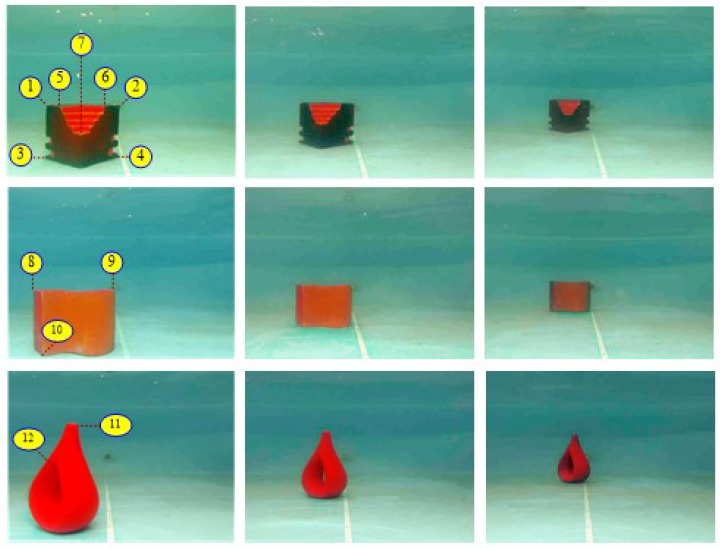
Underwater targets with observed points marked for distance measurement, the distances are about 1 m, 1.5 m and 2 m from left to right.

We have given a complete vision system in our work and successfully tested it on targets around 1-m distance in a reasonably clear water pool. Now the system can stably capture the images underwater for more than one hour and processes a pair of left and right images with a total latency of about 100 milliseconds. But the system does have a restriction on measuring distance, because we currently focus on improving the stereo matching, without thoroughly considering the problem of camera calibration caused by light refraction in air-glass-water transitions, which is another important issue. In our current work, following the common practice, we adopt a pinhole model with the parameters obtained by an in-water calibration using a checkerboard at about 1 m away. According to the work in Reference [7], the distance measurement accuracy will descend if the target is not close to the distance at which the camera was calibrated. To find the maximum distance of our system, we then test it on other three underwater targets at different distances (1-m, 1.5-m and 2-m distances). The images with the observed points marked are given in Figure 14 and the measured distances of these points are given in Table 7. Table 7 also gives the true distance values of the observed corner points when the targets are put about 1 m away and the true values of these points should be added 50 cm and 100 cm when the targets are measured at 1.5 m and 2 m, respectively.

From Table 7, it can be seen that the proposed method has the lowest distance measurement error rate (mean error of 0.82%, about 0.85 cm) at 1-m distance, which is the distance at which the camera was calibrated. The error rate (mean error of 1.533%, about 2.5 cm) becomes a little bigger at 1.5-m distance. However, when the distance comes to 2 m, the error rate becomes much bigger (mean error of 5.260%, about 11 cm), which may not be acceptable. The main reason for this is that the pinhole model used in our system can only very well approximates an axial camera model around the distance at which the camera was calibrated [7]. Therefore, the working distance of the current system should be restricted around 1 m and within 2 m distance.

## 6. Conclusions

To deal with the difficulty of underwater target detection and 3D reconstruction in optical image, a binocular vision based underwater target detection and 3D reconstruction system is proposed in this paper. The left and right views of the valid target area are obtained by image preprocessing technologies, such as image denoising, color restoration, salient region segmentation and so on, which will help to reduce mismatch caused by noise and distortion.

Based on the improved semi-global matching algorithm and the least squares plane fitting method, the accuracy of the three-dimensional reconstruction of the targets are effectively improved, which is good for practical engineering applications. However, due to the pinhole model adopted for camera calibration in our research, the current system can only work well within 2 m distance. Therefore, it is necessary to do more research on the pinax model, which is more accurate and apply it to our system in the future work. Moreover, for the convenience of the experiments and the validation, the current work is carried out only in a reasonably clear water pool, its robustness in a more turbid water needs to be verified in the future research. 

## Figures and Tables

**Table 1:** Intrinsic parameters of the binocular vision system (in water, at 1 m distance).

View	Optical Geometry Parameters				Distortion Parameters				
	*f_x_*	*f_y_*	*u*_0_	*v*_0_	*k*_1_	*k*_2_	*p*_1_	*p*_2_	*k*_3_
**Left**	7.44 × 10^2^	7.46 × 10^2^	3.16 × 10^2^	2.22 × 10^2^	−2.35 × 10^−1^	5.22 × 10^−2^	−6.60 × 10^−4^	1.09 × 10^−3^	0
**Right**	7.40 × 10^2^	7.42 × 10^2^	2.77 × 10^2^	2.44 × 10^2^	−2.45 × 10^−1^	7.94 × 10^−2^	−6.26 × 10^−4^	7.94 × 10^−4^	0

**Table 2:** Intrinsic parameters of the binocular vision system (in the air, at 1 m distance).

View	Optical Geometry Parameters				Distortion Parameters				
	*f_x_*	*f_y_*	*u*_0_	*v*_0_	*k*_1_	*k*_2_	*p*_1_	*p*_2_	*k*_3_
**Left**	5.51 × 10^2^	5.53 × 10^2^	3.04 × 10^2^	2.20 × 10^2^	−3.60 × 10^−1^	1.62 × 10^−1^	−1.56 × 10^−3^	5.45 × 10^−4^	0
**Right**	5.50 × 10^2^	5.51 × 10^2^	2.74 × 10^2^	2.44 × 10^2^	−3.66 × 10^−1^	1.79 × 10^−1^	−1.55 × 10^−3^	4.49 × 10^−4^	0

**Table 3:** External parameters of the binocular vision system (in water and in the air, at 1 m distance).

	In Water	In the Air
*R*	$\left[\begin{array}{ccc} 0.99 & 6.36\times 10^{-3} & -3.74\times 10^{-3}\\ -6.43\times 10^{-3} & 0.99 & 1.88\times 10^{-2}\\ 3.62\times 10^{-3} & -1.88\times 10^{-2} & 0.99\; \end{array}\right]$	$\left[\begin{array}{ccc} 0.99 & -3.72\times 10^{-2} & 2.15\times 10^{-2}\\ 3.79\times 10^{-2} & 0.99 & -3.42\times 10^{-2}\\ -2.02\times 10^{-2} & 3.49\times 10^{-2} & 0.99 \end{array}\right]$
*T*	[−45.54 0.69 −1.82]	[−119.53 −5.88 2.62]
*Q*	$\left[\begin{array}{cccc} 1 & 0 & 0 & -2.62\times 10^{2}\\ 0 & 1 & 0 & -2.22\times 10^{2}\\ 0 & 0 & 0 & 7.67\times 10^{2}\\ 0 & 0 & 2.19\times 10^{-2} & 0 \end{array}\right]$	$\left[\begin{array}{cccc} 1 & 0 & 0 & -2.94\times 10^{2}\\ 0 & 1 & 0 & -2.33\times 10^{2}\\ 0 & 0 & 0 & 5.82\times 10^{2}\\ 0 & 0 & 8.35\times 10^{-3} & 0 \end{array}\right]$
err/pixel	0.173	0.155

**Table 4:** Fitting parameters of photoreceptor curve.

	A		B		C		D	
	Left	Right	Left	Right	Left	Right	Left	Right
**r**	0.0001	0.0001	−0.0349	−0.0309	5.7850	5.3128	−134.8712	−74.8997
**g**	−0.0001	−0.0002	0.0378	0.0612	−2.6504	−4.0552	45.2350	64.2145
**b**	−0.0000	−0.0002	0.0166	0.0971	−0.2026	−10.5140	−98.9514	351.4542

**Table 5:** The fitting parameters of the disparity plane templates.

Plane No.	Map 1			Map 2			Map 3		
	*a*	*b*	*c*	*a*	*b*	*c*	*a*	*b*	*c*
**1**	−0.015	0.024	33.135	−0.017	0.359	76.050	0.020	0.014	59.434
**2**	0.066	0.309	6.412	−0.043	0.023	89.671	0.185	0.494	−55.594
**3**	−0.096	0.4856	14.104	−0.177	0.222	108.943	−0.391	0.480	35.146
**4**	0.017	0.086	28.928	−0.015	0.120	106.618	−0.031	0.271	52.872
**5**	−0.057	0.315	37.055	−0.187	0.218	113.908	−0.002	0.268	42.797
**6**	−0.101	0.350	44.062	−0.051	0.460	3.560	−0.002	0.250	48.037

**Table 6:** Data of distance measurement experiments for underwater targets (unit: cm).

No.	True Value	AD-Census	FCVF	ARWR	SGM	Ours
**1**	167.096	165.722	168.924	152.695	155.410	156.804
**2**	150.310	149.433	164.940	150.074	139.312	154.041
**3**	163.845	170.572	172.253	168.924	197.555	166.511
**4**	50.912	47.590	47.442	46.539	53.170	49.326
**5**	70.200	74.339	74.962	68.152	67.053	71.222
**6**	153.310	155.410	152.695	150.074	146.921	154.722
**7**	142.698	148.166	137.127	142.143	155.410	145.092
**8**	172.380	137.127	142.143	162.638	139.312	169.744
**9**	139.237	172.253	168.924	144.493	172.253	138.759
**10**	137.437	137.127	168.924	137.127	139.312	137.127
**Mean Error**		6.752%	9.564%	3.553%	10.114%	2.320%

**Table 7:** Distance measurement experiments for underwater targets at different distances (unit: cm).

No.	True Value (at 1 m)	Measured (at 1 m)	Measured (at 1.5 m)	Measured (at 2 m)
**1**	126.110	124.960	170.434	213.432
**2**	126.510	125.460	173.511	215.253
**3**	126.251	127.342	170.552	213.721
**4**	126.680	125.978	173.822	214.737
**5**	119.012	118.203	165.351	206.198
**6**	119.112	118.250	166.354	207.935
**7**	105.420	104.560	156.326	194.759
**8**	106.450	105.881	154.442	194.275
**9**	105.300	106.566	156.500	195.964
**10**	100.011	101.052	148.911	189.896
**11**	100.120	100.800	151.120	189.742
**12**	97.200	98.100	146.115	187.613
**Mean Error**		**0.821%**	**1.533%**	**5.260%**
